# Comparison of condylar morphology changes and position stability following unilateral and bilateral sagittal split mandibular ramus osteotomy in patients with mandibular prognathism

**DOI:** 10.1186/s13005-019-0202-z

**Published:** 2019-07-11

**Authors:** Han Lin, Yifan He, Yifan Feng, Fang Huang

**Affiliations:** 1 Department of Oral and Maxillofacial Surgery, Guanghua School of Stomatology, Hospital of Stomatology, Sun Yat-sen University, Guangdong Provincial Key Laboratory of Stomatology, Guangzhou, China; 2 Department of Orthodontics, Guanghua School of Stomatology, Hospital of Stomatology, Sun Yat-sen University, Guangdong Provincial Key Laboratory of Stomatology, Guangzhou, China; 3 Department of Prothodontics, Guanghua School of Stomatology, Hospital of Stomatology, Sun Yat-sen University, Guangdong Provincial Key Laboratory of Stomatology, Guangzhou, China; 4 Department of Paediatric Dentistry, Guanghua School of Stomatology, Hospital of Stomatology, Sun Yat-sen University, Guangdong Provincial Key Laboratory of Stomatology, Fang Huang, No.56 Lingyuan Xi Road, Guangzhou, Guangdong Province People’s Republic of China 510055

**Keywords:** Facial asymmetry, Mandibular prognathism, Unilateral sagittal split ramus osteotomy, Condylar surface morphology, Temporomandibular joint

## Abstract

**Background:**

Unilateral sagittal split ramus osteotomy (USSRO) is not widely used given the postoperative instability caused by the inevitable rotation of the mandibular segment during surgery. However, the influence of mandibular movement on the condylar morphology and position stability has not been completely explored. The aim of the study was to quantitatively evaluate the effect of USSRO on the condylar surface morphology changes and postoperative stability in patients with mandibular lateral prognathism and compare these findings with the classic bilateral sagittal split ramus osteotomy (BSSRO).

**Patients/methods:**

This was a retrospective study involving 134 patients with mandibular lateral prognathism who received USSRO (*n* = 56) and BSSRO (*n* = 78) surgery. Here, cone beam computed tomography (CBCT) was performed before surgery (T0), immediately after surgery (T1), and 1 year postoperatively (T2). Differences of condylar sizes, condylar surface deviation, and mandibular positioning parameters (dental midline deviation, SNB, SN-MP) were calculated from T0 to T2. Comparisons were performed at the deviated side or nondeviated side of condyles between the USSRO and BSSRO groups. The relation between the dental midline deviation and condylar surface morphology changes from T0 to T2 were investigated.

**Results:**

Condylar surface morphology changes at the deviated side of temporomandibular joint (TMJ) before and 1 year after the surgery were significantly different between the USSRO and BSSRO groups. The dental midline deviation was related to the changes of condylar volume, surface size and surface deviation at the deviated side of TMJ in patients following USSRO. No significant difference was noted between the USSRO and BSSRO groups for postoperative condylar surface morphology changes at the nondeviated side. In both groups, significant differences between T0 and T1 and no significant difference between T1 and T2 were noted for all of the mandibular positioning parameters.

**Conclusions:**

Both BSSRO and USSRO exhibit favorable postoperative stability in the correction of mandibular prognathism. After USSRO surgery, condylar surface changes occurred at the deviated side of the TMJ, and the dental midline deviation was closely related to the changes of condylar surface morphology. USSRO represents a stable alternative for minor asymmetric mandibular prognathism correction with the advantages of reduced operating time and surgical trauma.

## Introduction

Deviated mandibular prognathism is one of the common craniofacial deformities with a lateral shift of the mandible midline [1]. It is a three-dimensional dentofacial deformity that involves asymmetry of the mandible, maxilla and chin [2]. The geometric complexity of the facial asymmetry makes it an extremely challenging prospect in orthognathic surgery.

Bilateral sagittal split mandibular ramus osteotomy (BSSRO) is considered a standard procedure in the correction of facial asymmetry [3]. By completely separating the distal mandible segment from bilateral proximal segments, it allows the tooth-bearing segment to move freely to achieve midline alignment and also ensures that the condyles in the proximal segments is seated in the neutral position of glenoid fossa with minimal temporomandibular joint (TMJ) tension [4]. However, some disadvantages of BSSRO include neurovascular injury, relapse, unintentional fractures, and malocclusion.

In recent years, unilateral sagittal split ramus osteotomy (USSRO) has been applied for correction of asymmetric mandibular prognathism and yielded favorable outcomes [5]. USSRO has the advantages of reducing operation time and the incidence of postoperative complications. In contrast to BSSRO, USSRO operates on only one side of the mandible and requires a rotation of the tooth-bearing segment to align the lower and upper dental midline, which would inevitably rotate the mandibular segment [5]. The key point to assess a successful operation is whether the movement of the mandibular segment during USSRO would affect the postoperative stability. Only if long-term postoperative stability is guaranteed, unilateral surgery could be accepted as a standard procedure for correcting facial asymmetry. Although many studies have proved the validity and postoperative stability of BSSRO, limited data about USSRO are available.

With regard to the relation of the condylar rotation and the postoperative stability, different scholars have different views. Some believe that the displacement of condyles during SSRO has a significant influence on early postoperative relapse and TMJ dysfunction, such as TMJ pain, clicking sounds, disc displacement and condylar resorption [6]. In contrast, some researchers considered that although three-dimensional changes in condylar head position are observed in patients post SSRO, there are no significant changes that would clinically affect the patients [7]. Therefore, larger sample size evaluation of patients following USSRO surgery and longer postoperative follow-up study are needed.

Whether the mandible movement during surgery would cause abnormal morphology changes in TMJ condyles is another key to determine whether USSRO can be regarded as a standard surgical procedure for the correction of deviated mandibular prognathism. Orthognathic surgery-induced changes of condylar location will impart physical stress on the condylar surface and subsequently cause condylar remodeling of the TMJ structure. If the stress exceeds the adaptation ability of TMJ, joint dysfunction will occur and ultimately lead to postoperative relapse.

With the development of imaging technology, numerous CBCT-based analysis methods have been introduced to assess condylar remodeling. For comprehensive evaluation of condylar surface changes, three-dimensional (3D) reconstructed models and a method of superimposition over the ramus region are necessary [8]. da Motta et al. used a 3D CBCT overlapping technique to evaluate mandibular anatomy and condylar location, proving its effectiveness [9]. An, S., et al. evaluated TMJ remodeling by the superimposition of the 3D reconstructed condylar models in patients with BSSRO or two jaw surgery and found that condylar surface changes before and after the surgery were significantly different [10]. In addition, numerous studies revealed postoperative condylar morphological changes following orthognathic surgery [11]. However, to the best of our knowledge, no study has assessed the effect of unilateral osteotomy on the condylar surface morphology and mandibular positioning changes in patients with lateral mandibular prognathism using 3D-reconstructed models and condylar superimposition.

The objective of this study was to quantitatively evaluate TMJ condylar surface morphology changes and postoperative stability following USSRO in patients with mandibular lateral prognathism compared with the classic BSSRO technique. In addition, the purpose of the study was to determine the relationship between the amount of midline deviation and condylar surface morphology changes after SSRO surgery.

## Materials and methods

### Ethics statement

The institutional ethics board at the Sun Yat-sen University approved the study protocol of this retrospective study. All patients in the study group consented to be a part of trial after clinical briefing on methodology, and they were informed of the details of the study. Informed consent was signed by patients. The institutional ethics board of the Hospital of Stomatology, Sun Yat-sen University, approved the reconstruction models.

### Patients and group selection

The patients who underwent USSRO or BSSRO surgery at the department of oral and maxillofacial surgery, Hospital of Stomatology, Sun Yat-sen University from January 2005 to December 2017 were enrolled in the study. All of the surgeries were performed using Hunsuck modified SSRO surgical procedure [12].

The following inclusion criteria of these patients were applied for the study population: patients with asymmetric mandibular prognathism; a deviated lower chin and lower dental arch midline; without temporomandibular joint disorders, such as joint clicks or noises; without progressive condylar resorption; and with or without a requirement for bimaxillary surgery beginning with a LeFort I osteotomy. All of the patients were received presurgical orthodontic treatment.

The exclusion criteria for both groups were as follows: asymmetry with an origin that was not lateral mandibular prognathism (such as active condylar hyperplasia, condylar degeneration); asymmetry caused by mandibular fracture or trauma; patients with incomplete clinical and imaging examination data.

### Parameters of evaluation

For each patient, an integral preoperative and postoperative assessment, including lateral cephalometric radiographs, panoramic radiographs, and CBCT; standard clinical and intraoral photographs; and a cephalometric evaluation were performed (before surgery: T0, immediately after surgery: T1, and 1 year postoperatively: T2).

**Fig. 1:**
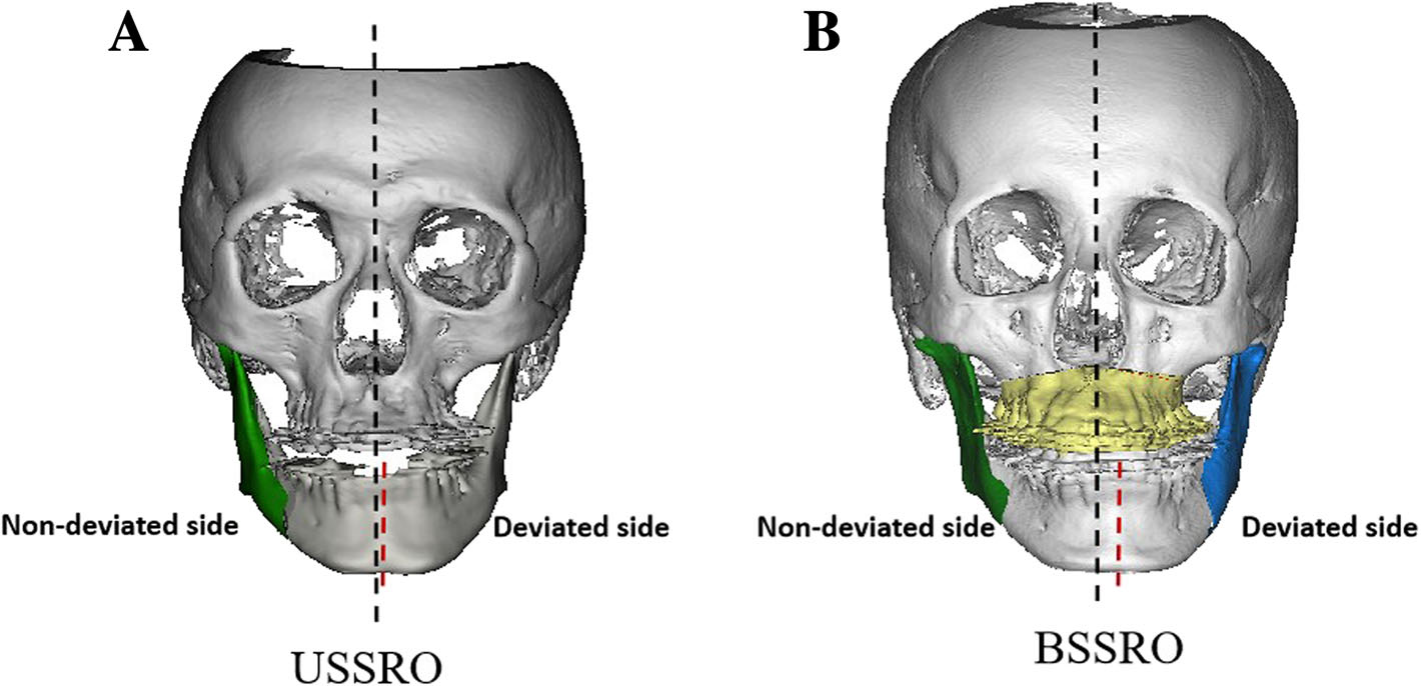
The 3D constructed models of mandibular asymmetry patients. The black dot line indicates the facial sagittal plane, and the red dot line indicates the lower dental arch midline: A. USSRO, the deviated side is the nonsurgical side; B. BSSRO, the deviated side is the shortened side

With regard to the evaluation of TMJ condylar morphology, condylar sizes (volume and surface size) and condylar surface deviation at T0 and T2 were measured. The differences between these values were calculated. For evaluation of the effect of different surgical procedures on the specific side of the TMJ condyle, deviated (shortened side, for USSRO, that is the nonsurgical side) and nondeviated sides (lengthened side, for USSRO, that is the surgical side) of TMJ subject to BSSRO and USSRO surgery were compared (Fig. 1).

### The condylar volume and surface size calculation (T0-T2)

**Fig. 2:**
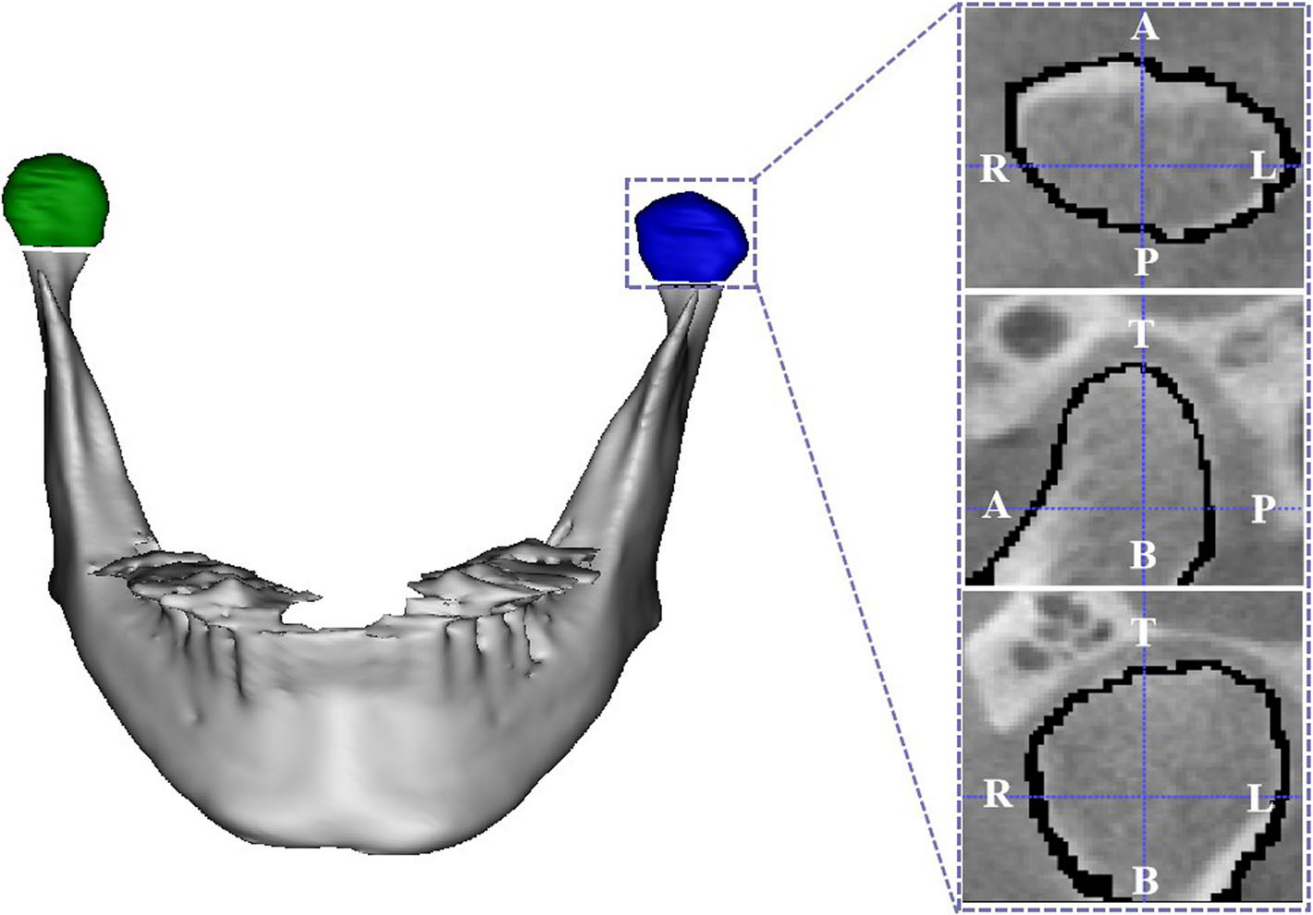
The superior, inferior and lateral limits of the 3D constructed models of mandibular condyles

CBCT data sets were acquired with a DCT Pro CBCT (Vatech, Co., Ltd., Hwasung, Korea) using the following scanning parameters: 90 kVp, 24 s, 4 mA, voxel size 0.4 mm and field of view 20 × 19 cm. The images covered the area from the upper orbits rim to the inferior border of the mandibular body. The gross data and slices were imported and used to reconstruct 3D models with an interactive image system (Materialise’s interactive medical image control system, Mimics, 14.0; Materialise, Leuven, Belgium). The upper and lower limits of the condyle were defined according to Tecco, S., et al. (Fig. 2) [13]. Volumetric (mm^3^) and surface size measurements (mm^2^) were made for condyles on two sides at the T0 and T2 stages in the USSRO and BSSRO groups using the Mimics™ automatic function.

### Surface morphology deviation evaluation

**Fig. 3:**
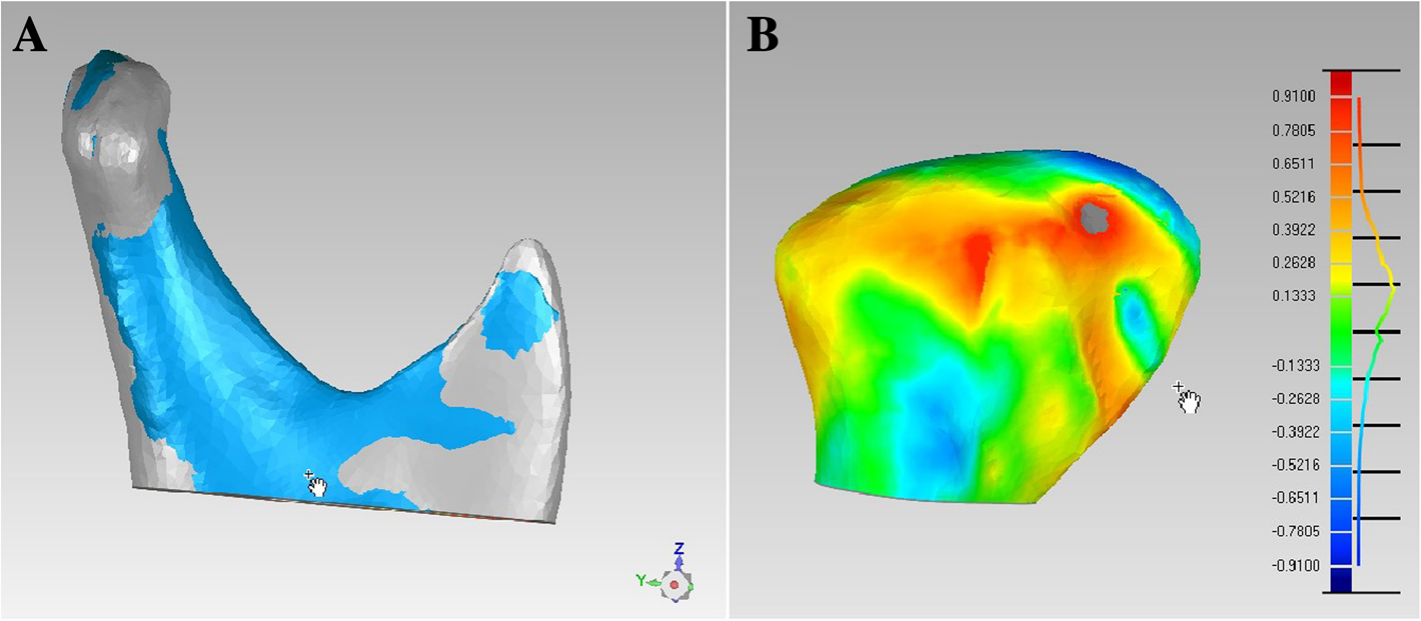
Average surface deviation measurement of the superimposed pre- and postsurgery mandibular condyles images: A. Superimposed pre- and postsurgery condyles models over three registration areas; B. Calculation of surface deviation of the superimposed condylar heads

To investigate the condylar surface morphology changes, pre- and postoperative reconstructed 3D models were superimposed over three registration areas (condylar neck, mandibular notch, and posterior border of ramus) [14]. The surface displacements of the heads were calculated using Geomagic Studio 12.0 (Geomagic, America) using the 3D surface-to-surface matching (best fit method), which employs a least-mean-squared algorithm. According to the coordination between the registration areas, the reconstructed images were automatically fitted. Given that the procedure is fully automated, the influence of the observers’ variability on the accuracy of measurements is completely eliminated. Then, the averages of the absolute value of the differences between the pre- and postoperative 3D condylar surface models were calculated (Fig. 3).

### Postoperative positioning stability evaluation of mandibular segments

**Fig. 4:**
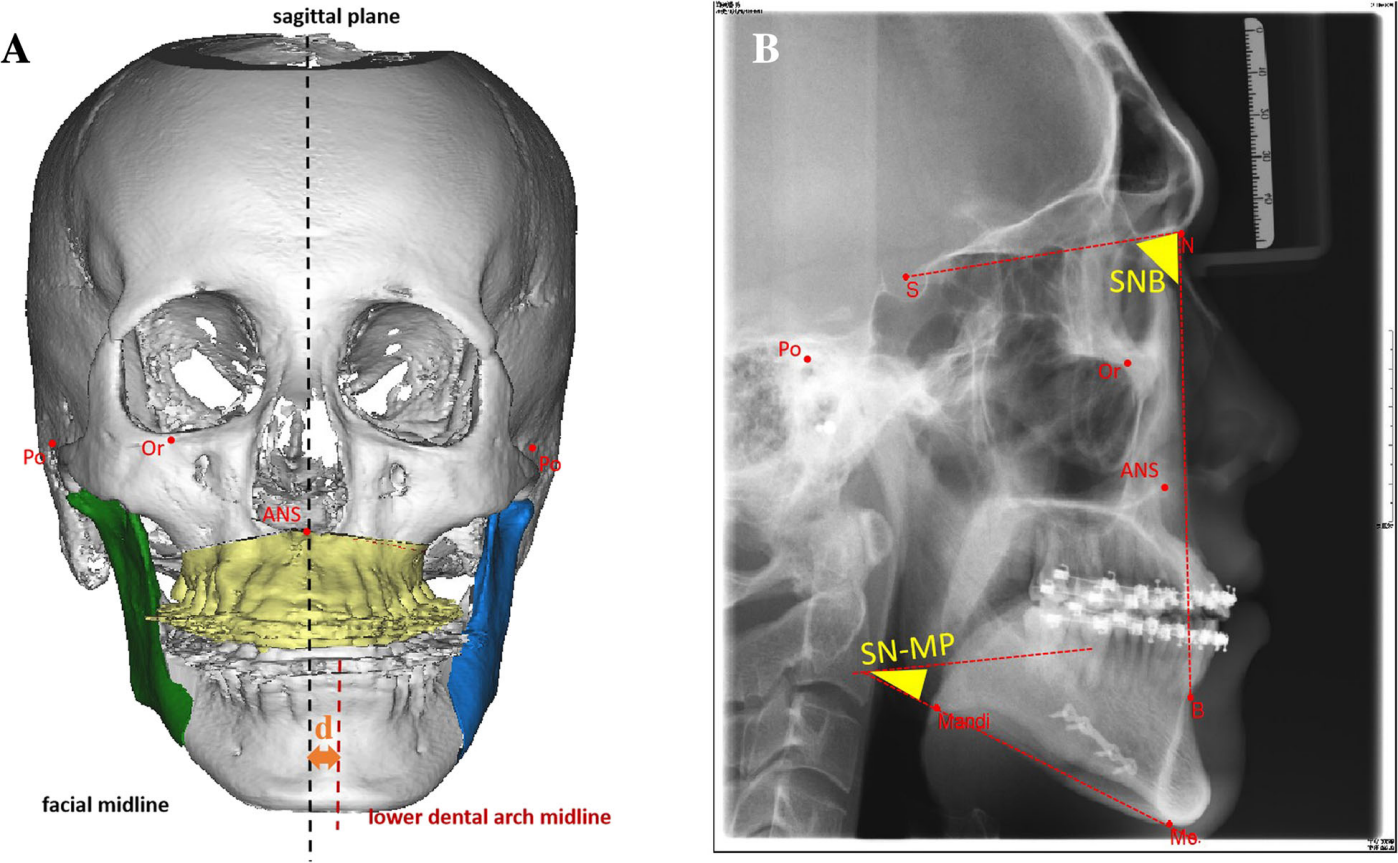
The clinical and cephalometric parameters of postoperative positioning stability evaluation of mandibular segments: A. lower dental midline deviation; B. SNB angle and SN-MP angle

To study the postoperative positioning stability of mandibular segments following USSRO and BSSRO from T0 to T1 and T1 to T2, related clinical and cephalometric parameters were selected as follows (Fig. 4 and Table 1):

1. Lower dental midline deviation, i.e., the deviation of lower dental midlines was defined as the horizontal distance between the facial sagittal plane and mesial contact points of mandibular central incisors, was used to evaluate the horizontal positioning stability of mandibular segment 1 year after surgery.
2. SNB, which is the position of mandible to skull base, was used to evaluate the sagittal positioning stability of mandibular segment immediate after surgery and 1 year after surgery.
3. SN-MP, the cant of lower border of mandible to skull base, was used to evaluate the vertical positioning stability of mandibular segment 1 year after surgery.

**Table 1:** Landmarks and measurements used in this study

Landmarks	Definition
Landmarks and planes:	
Sella (S.)	The point representing the midpoint of the pituitary fossa (sella turcica)
Nasion (N.)	The most anterior point of the frontonasal suture
Supramentale (B.)	The point at the deepest midline concavity on the mandibular symphysis between infradentale and pogonion
Orbitale (Or)	Mid-point of the infraorbital margin
Anterior nasal spine (ANS)	Tip of the anterior nasal spine
Porion (Po)	Superior point of the external auditory meatus
Mandibular Plane (MP)	The tangent line to the lower border of the mandible through gnathion
Anterior cranial base (SN)	The line connecting the center of Sella turcica and Nasion
Axial plane	Plane containing both porions and the right Or
Coronal plane	Plane perpendicular to axial plane that includes both Po
Sagittal plane	Plane perpendicular to axial and coronal planes that includes ANS
Measurements	
Dental midline deviation	The deviation of dental midlines was defined as the horizontal distance between the facial sagittal plane and mesial contact points of mandibular central incisors as measured on the 3D reconstructed models
SNB	the position of mandible to skull base
SN-MP	The cant of lower border of mandible to skull base

### Evaluation of TMJ function stability after USSRO and BSSRO

To study the postoperative TMJ function stability following USSRO and BSSRO from T0 to T1 and T1 to T2, related TMJ clinical examinations such as TMJ pain, clicking and maximum mouth opening at T0, T1 and T2 were measured and compared respectively. Maximum mouth opening was measured as the distance between the upper and lower incisors at maximum active mandibular depression. Evaluation of TMJ function was conducted by collection of the patient reports and existing clinical records.

### Statistical analysis

The data were presented as the mean ± standard deviation. Data were processed and analyzed using SPSS 16. 0 (SPSS Inc., Rainbow Technologies, Chicago). The independent two sample t-test was used to calculate the morphological changes and positioning stability on each side of the condyle between the USSRO and BSSRO group. A paired t-test was used to calculate the TMJ function and positioning stability within each group. *P* < 0.05 was considered significant. To analyze the correlation between the amount of dental midline deviation and condylar surface morphology changes, relevant Pearson correlation coefficients were calculated. In the study, the measurements were performed by the same experienced clinical doctor (DJ), and the consistency of the measurement method was assessed by one-way random intraclass correlation coefficients (ICCs). ICC values were all greater than 0.9, demonstrating the high reliability of these measurements.

## Results

Of the 134 patients we reviewed, 56 patients (23 males/33 females, mean age 26.4 years) underwent USSRO surgery with or without Lefort I osteotomy, and 78 patients (37 males /41 females, mean age 23.1 years) underwent BSSRO surgery. In the USSRO group, there was an average mandibular dental deviation of 4.3 mm (range from 2.7 mm to 8.3 mm) before surgery. In the BSSRO group, there was an average mandibular dental deviation of 5.1 mm (range from 2.8 mm to 13.5 mm) before surgery. All of the patients had a negative ANB angle and underwent mandibular segment setback osteotomy (Table 2).

**Table 2:** Clinical data summary of patients enrolled in the present study

Group	Case number	Age distribution	Sex distribution	Direction of movement	Average lower arch midline deviation (mm)	Average ANB angle (degree)
USSRO	56	26.4 ± 5.7 y (range 18.1–37.8 y)	23 males/33 females	setback	4.3 ± 1.3 (range 2.7–8.3)	−3.6 ± 0.5
BSSRO	78	23.1 ± 4.9 y (range 17.0–35.1 y)	37 males /41 females	setback	5.1 ± 1.9 (range 2.8–13.5)	− 3.7 ± 0.5

*USSRO* unilateral sagittal split ramus osteotomy, *BSSRO* bilateral sagittal split ramus osteotomy

In the USSRO group, the average condylar volume changes of the deviated and nondeviated side of TMJ (from before surgery (T0) to a year after surgery (T2)) were 98.3 ± 29.3 mm^3^ and 58.7 ± 9.7 mm^3^, respectively. In the BSSRO group, the average volume changes of the deviated and nondeviated side of TMJ (from T0 to T2) were 71.6 ± 11.9 mm^3^ and 59.3 ± 8.4 mm^3^, respectively. A significant difference was observed between the USSRO and BSSRO groups on the deviated side of the TMJ (*P* < 0.01).

Regarding the comparison of surface size between the USSRO and BSSRO groups, a significant difference was found on the deviated side of TMJ between T0 and T2 (*P* < 0.05). For the evaluation of the average surface deviation, a significant difference was also noted between the USSRO and BSSRO groups on the deviated side of TMJ (*P* < 0.01). No significant differences in the morphology parameters were noted on the nondeviated side (Table 3).

**Table 3:** Comparison of condylar size and morphology changes from preoperative to 1 year after surgery (T0-T2) between the USSRO and BSSRO groups

T0-T2 TMJ condylar change	Condylar volume change (mm^3^)		Condylar surface size change (mm^2^)		Average surface deviation of TMJ (mm)	
	Deviated side	Nondeviated side	Deviated side	Nondeviated side	Deviated side	Nondeviated side
USSRO	98.3 ± 29.3	58.7 ± 9.7	80.4 ± 18.9	40.2 ± 8.3	0.25 ± 0.05	0.15 ± 0.03
BSSRO	71.6 ± 11.9	59.3 ± 8.4	51.1 ± 13.8	39.7 ± 11.0	0.14 ± 0.03	0.14 ± 0.03
*P* value	< 0.01^**^	0.33	0.011^*^	0.09	0.001^**^	0.156

T0: preoperative, T2: 1 year after surgery, ^*^: *P* < 0.05, ^**^: *P* < 0.01

In the evaluation of dental midline correction across the different time intervals, the BSSRO group exhibited a significant difference between T0 and T1 (*P* < 0.01), but no significant difference was noted between T1 and T2 (*P* = 0.235). The same evaluation in the USSRO group revealed a significant difference between T0 and T1 (*P* < 0.01) but no significant difference between T1 and T2 (*P* = 0.163). Regarding the SNB angle and SN-MP angle, also significant differences were noted between T0 and T1 within each group, whereas no significant difference was found between T1 and T2. These results indicated that patients in both groups exhibited satisfactory asymmetry correction and postsurgical stability without obvious mandibular segment rotation (Table 4).

**Table 4:** Comparison of dental midline deviation, SNB angle, and SN-MP angle between the different time points in the USSRO and BSSRO groups: T0 (preoperative), T1 (immediately after surgery), T2 (1 year after surgery)

Parameters	Dental midline deviation (mm)		SNB changes (°)		SN-MP changes (°)	
	T0-T1	T1-T2	T0-T1	T1-T2	T0-T1	T1-T2
USSRO	3.9	0.4 ± 0.1	5.4 ± 1.6	1.5 ± 0.5	2.3 ± 0.8	1.0 ± 0.6
BSSRO	4.8	0.4 ± 0.1	5.6 ± 2.7	1.4 ± 0.4	3.3 ± 0.9	2.0 ± 0.7
*P*-value within USSRO	< 0.01^**^	0.163	< 0.01^**^	0.438	< 0.01^**^	0.232
*P*-value within BSSRO	< 0.01^**^	0.235	< 0.01^**^	0.419	< 0.01^**^	0.147
*P*-value between USSRO and BSSRO	< 0.01^**^	0.062	< 0.01^**^	0.462	0.267	0.082

T0: preoperative, T1: immediately after surgery, T2: 1 year after surgery, ^**^: *P* < 0.01

To uncover the relationship between the amount of dental midline deviation and the condylar surface morphology changes, a Pearson correlation analysis was performed. After surgery, significant coefficients were observed for the amount of dental midline deviation and the morphology changes of the deviated side following USSRO surgery (volume change, *r* = 0.784, *P* < 0.01; surface size change, *r* = 0.717, *P* < 0.01; average surface deviation, *r* = 0.782, *P* < 0.01). However, no significant coefficients were noted for the other groups (Table 5).

**Table 5:** Correlation between extents of dental midline deviation and condylar surface morphology changes from preoperative to 1 year after surgery (T0-T2)

	USSRO		BSSRO	
	Pearson Correlation Coefficient	*P*-value	Pearson Correlation Coefficient	*P-*value
Volume change on deviated side	0.784	< 0.01^**^	0.060	0.601
Volume change on nondeviated side	0.173	0.20	0.055	0.631
Surface size change on deviated side	0.717	< 0.01^**^	0.014	0.920
Surface size change on nondeviated side	0.046	0.73	0.058	0.614
Average surface deviation on deviated side	0.782	< 0.01^**^	0.242	0.033
Average surface deviation on nondeviated side	0.115	0.40	0.145	0.205

T0: preoperative, T2: 1 year after surgery, ^**^: *P* < 0.01

The evaluation results of TMJ function stability were presented in Table 6. For USSRO group, the average values of maximum mouth opening were 48.1 mm, 24.8 mm, and 45.4 mm for T0, T1 and T2, respectively. There were significant differences between T0-T1 and T1-T2 (*P* < 0.05, *P* < 0.01). Similar results were observed in the BSSRO group, the average values of maximum mouth opening were 49.2 mm (T0), 21.6 mm (T1), and 48.4 mm (T2), respectively.

**Table 6:** Evaluation of maximum mouth opening after USSRO and BSSRO

	USSRO			BSSRO		
	T0	T1	T2	T0	T1	T2
Mean ± SD (mm)	48.1 ± 6.2	24.8 ± 8.3	45.4 ± 5.5	49.2 ± 7.1	21.6 ± 8.1	48.4 ± 8.8
*P* (T0-T1)	0.038^*^			0.001^**^		
*P* (T0-T2)	0.468			0.078		
*P* (T1-T2)	0.005^**^			0.001^**^		

T0: preoperative, T1: immediately after surgery, T2: 1 year after surgery, ^*^: *P* < 0.05, ^**^: *P* < 0.01

As for the evaluation of the incidence rate of TMJ pain in USSRO group, 19.6% (11/56) of the patients reported joint pain at T0, while at T1 and T2, the proportion were 48.2% (27/56) and 14.3% (8/56). In the BSSRO group, the incidence rates of TMJ pain were 24.4% (19/78, T0), 41.0% (32/78, T1) and 14.1% (11/78, T2), respectively. The incidence rates of TMJ clicking of patients in USSRO and BSSRO were 26.8% (15/56, T0), 32.1% (18/56, T1), 35.7% (20/56, T2) and 29.5% (23/78, T0), 21.8% (17/78, T1), 26.9% (21/78, T2), respectively.

## Discussion

Currently, the research hotspots in orthognathic surgery include operation safety, in particular the protection of alveolar nerve; postoperative complications, such as the bony healing; long-term postoperative stability of skeletal movements and the stability of TMJ structure and function. In recent years, USSRO has gradually been used to correct lateral deviation of the mandible [15]. Increasing research interests have been focused on whether USSRO can be applied as an effective operation for the correction of mandibular deviation.

The postoperative stability of USSRO is the first concern, as it is closely related to the rotation of the articular condyle during USSRO. Some studies have been conducted to analyze the movement pattern of joints following SSRO surgery. Hu et al. reported that the condyles were displaced posteriorly and rotated anteriorly in 22 patients who underwent mandibular advancement surgery using SSRO [16]. Some papers indicated that the location changes of the condylar head during orthognathic surgery caused condylar resorption and led to recurrence [17, 18]. However, Kim, M.I., et al. noted that although positional changes in the condylar head are observed post USSRO in 3 cases during 6-month follow-up, there are no significant changes that would clinically affect patients [7]. The postoperative stability of patients with USSRO requires studies with larger sample size and longer follow-up.

TMJ constantly undergoes a remodeling process as responses to pressure stimuli during mandibular movement [19]. Joint function can be maintained as long as the changes of condylar position are within its physiological adaptive capacity. Given that changes in the axial direction of the TMJ condyles do not indicate postoperative stability, additional indicators are needed to assess the true postoperative outcome. In the present study, we selected three cephalometric parameters for post USSRO mandibular positioning evaluation from horizontal, sagittal and vertical dimensions. No significant differences between T1 and T2 were noted in the measurement of deviated dental midline, SNB and SN-MP in both of the BSSRO and USSRO groups. These results indicate good postoperative stability with at least 1 year of follow-up in both groups. Other studies also demonstrate that changes in the joint can maintain postoperative stability as long as it is within the joint adaptive capacity. Farina, R., et al. observed 14 patients with USSRO and reported that no patient showed signs or symptoms of temporomandibular joint dysfunction during the 1-year postoperative follow-up [15]. Wohlwender, I., et al. conducted a study on 23 patients to evaluate the long-term clinical and radiological effects on the TMJ following USSRO. Two years postsurgery, no significant differences were noted compared with the literature on BSSRO, and no patient displayed functional alterations on the side that was not subject to the operation [5].

Another important point regarding the maintenance of the joint function is that the morphology changes of joint surface morphology are within the capacity of joint self-regulation [20, 21]. Condylar surface morphology changes, such as joint resorption after orthodontic surgery, are common in clinical practice. With advances in anthropometry techniques, various studies attempt to objectively quantify TMJ with CBCT. For assessment of condylar remodeling, several CBCT-based analysis methods have already been introduced [22, 23]. However, most of the reports are limited to the linear measurement of the condyle, such as lengths, angles and vectors. Saccucci et al. demonstrated that the optimum size or volume of the mandibular condyle are indicative and predictive of a precise clinical situation [24]. In the study, we measured the volume and surface size changes of condyles in the USSRO and BSSRO groups and observed significant differences at the deviated side of TMJ before and 1 year after the surgery between the two groups.

For comprehensive evaluation of condylar surface changes, 3D reconstructed images and a method of superimposition are necessary. Ok, S.M., et al. and Park, S.B., et al. introduced CBCT superimposition methods for evaluating condylar surface morphology changes after orthognathic surgery in TMJ OA patients [14, 25]. However, there have been few 3D CBCT studies that quantitatively analyzed the condylar head surface changes in patients with USSRO. For model overlapping, the key is to identify the correct regions that are not subject to volumetric changes after orthognathic surgery for registration. Theo J.M. Hoppenreijs, et al. noted that regional superimposition of condyles with voxel-based registration on the coronoid process may particularly be interesting [26]. In the study, we superimposed reconstructed models with three registration areas (condylar neck, mandibular notch, and posterior border of ramus) according to the method of An, S.B. and Park, S.B. [10, 14]. It is found that condylar surface morphology changes at the deviated side of TMJ before and 1 year after the surgery were significantly different between the USSRO and BSSRO groups. Considering that the positional parameters of the mandible are not significantly changed, the result indicated that the condylar remodeling on the deviated side of TMJ in USSRO group is within the normal range of physiological resorption. Other authors used a margin of 6% as the threshold between physiological remodeling and resorption [14].

In the study, we demonstrate that the dental midline deviation was related to changes in condylar volume, surface size and surface deviation (*r* = 0.784, 0.717, and 0.782, respectively; all of the *P*-values were < 0.01). Our research is consistent with the study by An, S.B. [10], which found that an increase in the change in the condylar axis increased condylar resorption. Therefore, the inevitable rotation of the condyle of the major segment during correction of mandibular asymmetry by a USSRO cannot be neglected. It is necessary to clarify to what extent mandibular deviation can be corrected by USSRO with stable postoperative outcomes. In the study by Lee, S., et al., 7-mm unilateral mandibular movement after USSO would rotate the mandibular condyle 3° to 4° in nonosteotomy side without influencing TMJ function [27]. However, Beukes, J., et al. suggested that USSRO should only be performed for small mandibular asymmetries of less than 5 mm [2]. In the study, the average deviation of the lower arch midline is 4.3 mm, ranging from 2.7 mm to 8.3 mm, and all of the patients reported satisfying postoperative outcomes.

Some limitations do exist in this study. The work presented is still limited to a relative short-term follow-up study. One year follow up is not sufficient to show postoperative stability, also not with regard to TMJ-disorders. Future investigations will focus on long-term postoperative stability evaluation. Moreover, a prospective study with a larger sample size assessing the application of USSRO in the correction of more severe mandibular deviation is needed in the future.

## Conclusions

Both BSSRO and USSRO have demonstrated favorable postoperative stability in the correction of mandibular lateral prognathism. After USSRO surgery, condylar surface changes occurred at the deviated side of the TMJ, and the dental midline deviation was closely related to changes of condylar surface morphology. USSRO is a great alternative for minor asymmetric mandibular prognathism correction with the advantages of reduced operating time and surgical trauma with a stable outcome. Long-term postoperative stability evaluation of USSRO needs follow-up study.

## Data Availability

The datasets analyzed during the current study are available from the corresponding author on reasonable request.

## Abbreviations

3D: Three-dimensional
BSSRO: Bilateral sagittal split ramus osteotomy
CBCT: Cone beam computed tomography
ICC: Intraclass correlation coefficient
SSRO: Sagittal split ramus osteotomy
TMJ: Temporomandibular joint
USSRO: Unilateral sagittal split ramus osteotomy
